# On the Comparative Mortality of Pestilence and War

**Published:** 1854-10

**Authors:** 


On the Comparative Mortality of Pestilence and War. By Dr. Robertson.—Some interesting tables have issued from the Health Office, comparing the loss of life by war and pestilence. It appears that in 22 years of war there were 19,796 killed, and 79,709 wounded, giving an anual average of 899 killed, and 3623 wounded. In 1848-49, there were no fewer than 72,180 persons killed by cholera and diarrhœa in England and Wales, and 144, 360 attacked; 34,397 of the killed were able-bodied persons, capable of getting their own living! Besides these deaths from the great epidemic, 115,000 die annually, on an average, of preventible diseases; while 11,419 die by violence. Comparing the killed in nine great battles, including Waterloo, 4,740, with the number killed by cholera in London in 1848–49, 14,139, we find the difference of 9,399 in favor of war. In cholera visitations, twelve per cent., sometimes twenty per cent., of the medical men employed died. The London missionaries die as fast as those in foreign countries, and there are some districts in London which make the Mission Society ask themselves whether they have a right to send men into them. From the returns of twelve Unions it is found that 3,567 widows and orphans are chargeable to the cholera of 1848–49, entailing an expenditure of £121,000 in four years only. —*Edinburgh Monthly Journal*, May, 1854.

